# Recovery of Natural Antioxidants from Agro-Industrial Side Streams through Advanced Extraction Techniques

**DOI:** 10.3390/molecules24234212

**Published:** 2019-11-20

**Authors:** Radu Claudiu Fierascu, Irina Fierascu, Sorin Marius Avramescu, Elwira Sieniawska

**Affiliations:** 1 University of Agronomic Science and Veterinary Medicine, 59 Marasti Blvd., 011464 Bucharest, Romania; radu_claudiu_fierascu@yahoo.com (R.C.F.); sorin_avramescu@yahoo.com (S.M.A.); 2 National Institute for Research & Development in Chemistry and Petrochemistry – ICECHIM Bucharest, 202 Spl. Independentei, 060021 Bucharest, Romania; 3 Research Center for Environmental Protection and Waste Management, University of Bucharest, 36-46 Mihail Kogalniceanu Blvd., 050107 Bucharest, Romania; 4 Department of Pharmacognosy, Medical University of Lublin, 1 Chodzki, 20-093 Lublin, Poland

**Keywords:** food waste, extraction, re-utilization, active compounds, fruits, vegetables

## Abstract

Large amounts of agro-industrial waste are being generated each year, leading to pollution and economic loss. At the same time, these side streams are rich source of active compounds including antioxidants. Recovered compounds can be re-utilized as food additives, functional foods, nutra-/pharmaceuticals, cosmeceuticals, beauty products, and bio-packaging. Advanced extraction techniques are promising tools to recover target compounds such as antioxidants from agro-industrial side streams. Due to the disadvantages of classical extraction techniques (such as large amounts of solvents, increased time of extraction, large amounts of remaining waste after the extraction procedure, etc.), and advanced techniques emerged, in order to obtain more efficient and sustainable processes. In this review paper aspects regarding different modern extraction techniques related to recovery of antioxidant compounds from wastes generated in different industries and their applications are briefly discussed.

## 1. Introduction

In a general definition, agro-industrial side streams represent the organic and inorganic material generated as residues from different sectors: agriculture, livestock, dairy production, food, and beverage industry, etc. Due to the impressive amounts of wastes generated, the community of researchers is striving to find cost-competitive solutions for recovery of biologically active compounds from these materials. Agro-industrial side streams covers a wide range of products available as raw materials. These can serve as a source of other added value products, under the nomenclature of biowaste. Food waste are generated from different sectors of food industry, such as vegetables, fruits, milk, meat, fish, and wine production [1]. At a European level, up to 37 million tons from the food and drink industry are estimated as biowaste [2]. Another source for biowaste production is agro-forestry. These wastes consist of stems of the crop plants like wheat, rice, sugarcane, etc. [3]. Some statistics presented by different authors show that there are large amounts of wastes generated from agro-forestry sector: 709 million tonnes of wheat straw, 673 million tonnes of rice straw, 102 million tonnes of bagasse [4], 0.1 million tonnes of coir [5], and 2.96 million tonnes of solid olive waste [6].

These large amounts of wastes can produce pollution and economic loss [7] and causes landfilling to be no longer sustainable. With an estimation of increasing human population of 9.3 billion people in 2050 [2], the actual concern of scientific community is to transform these materials into added-value products for other industries.

Improper management of wastes leads to irreversible environmental issues, like global warming [8], just to name the most known and currently discussed environmental effect. The impact of nitrous oxide and methane generated during composting of different waste is estimated to be 20 times higher than carbon dioxide release in the next years [9]. In developed countries, the wastes are transformed in energy or commodity chemicals, while in less developed countries there is a gap for proper waste management due to insufficient infrastructure, policy framework and funds [10].

Antioxidants are found among several groups of natural compounds definitely worth to be recovered from agro-industrial waste and by-products (Table 1). They can be reached in peels, pomace, seed fractions, straws and other agro food wastes which are a rich source of phytochemicals like polysaccharides, dietary fibers, flavor compounds, etc. Polyphenols are among the largest classes of phytochemicals with antioxidant properties which are divided in hydroxybenzoic and hydroxycinnamic acids, anthocyanins, proanthocyanidins, flavonols, flavones, flavanols, flavanones, isoflavones, stilbenes, and lignans which can be extracted in different concentrations. Other antioxidant compounds which can be obtained are minerals, vitamins, carotenoids and low molecular weight antioxidants [11].

Recovered compounds can be re-utilized as food additives, functional foods, nutra-/pharmaceuticals, cosmeceuticals, beauty products and bio-packaging.

The field of active compounds extraction is the focus of an increasing number of studies in the last decades, as proven by the survey performed using several databases (ScienceDirect, Scopus, PubMed, Medline). The present paper is focused on recovery antioxidant compounds from edible oil industry, fruits and vegetable industry and wastes from other relevant industries (fish, meat, etc.)

The search was performed over the time period 2010–2019, using multiple key words: “antioxidant edible oil industry” (7170 articles), “antioxidant fruit industry” (16.832 articles), “antioxidant vegetable industry” (19.357 articles), “antioxidant fish industry” (13.735 articles) etc. Statistically, less than 1 third of the returned results were on the topic of our research (“recovery of antioxidants from agro-industrial side streams and potential applications”), rest of them being false positive responses. The criterion of acceptance was to provide information regarding extraction technologies, with emphasis on advanced technologies (such as pressurized liquid extraction, ultrasound-assisted extraction and electro technologies) used for the valorization of agro-industrial side streams and for recovery and application of antioxidant compounds (over 2000 papers). From all accepted papers, only those original studies presenting “green” advanced extraction techniques, published in top quality journals were selected. Whenever deemed necessary, in order to proper define the methods and techniques, the search was extended (up to the year 2000, selecting only the most recent studies).

In this review paper, aspects regarding different modern extraction techniques (conventional, electro-technologies, enzymatic) related to recovery of antioxidant compounds from wastes generated in different industries and their applications are briefly discussed. Some recent, comparative examples and details regarding solvents, parameters and the obtained results are also provided.

## 2. Recovery of Antioxidants from Agro-Industrial Side Streams

### 2.1. Recent Advances in Recovery of Antioxidants from Agro-Industrial Side Streams

**Figure 1:**
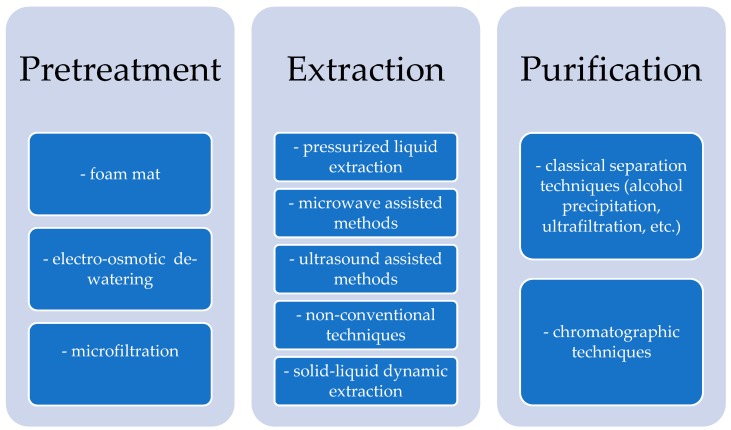
Flow chart of extraction process.

The use of agro-industrial side streams as a source of bioactive compounds cannot be performed without “green” advanced extraction techniques. The general flow chart of an extraction process is based on three general steps: pre-treatment, extraction, and purification (Figure 1), the yield of recovery of interest compounds being influenced by different parameters, as pre-treatment, solvent, temperature, agitation rate etc. The pre-treatment process is the first parameter considered. Pre-treatment based on heat is not applicable in antioxidants recovery, because it affects the process in a negative manner, with a simultaneous reduction of the phenolic concentrations and antioxidant capacity of the extracts [27]. Pre-treatment processes such as foam mat, electro-osmotic de-watering and micro-filtration are proposed by different authors, in order to keep biological activities unaltered and to remove microbes from vegetal material [28]. Alcohol precipitation is the most used technique for the separation of small particles (such as polyphenolic compounds or minerals) from macromolecules [29]. Ultra-filtration was applied for removing pectin and potassium from olive mill wastewater [30], while enzymatic treatment for extracting flavors and colors from plant materials [31].

The use of the solvent depends on the solubility and volatility of the target compounds. Phenols are easily solubilized in polar protic mediums [32], carotenoids are more liposoluble in polar aprotic or non-polar solvents [33]. The recovery of pectin and hemicelluloses requires complex solvent treatment with ethanol followed by alkali [29]. Several environmentally friendly solvents, alternatives to the use of classical organic solvents emerged in the last decades, such as ionic liquids, alcohols or terpenes, surfactant solutions or natural deep eutectic solvents. The extraction techniques applicable for the recovery of antioxidants from plant matrices are solid-liquid methods. Classical extraction method (such as Soxhlet extraction, percolation, maceration, hydro distillation, and steam distillation) are based on solid-liquid extraction with various solvents. They have significant drawbacks, especially in terms of long extraction time, relatively large quantities of organic solvents used and low yields of recovered target compounds [34]. Advanced methods are considered pressurized liquid extraction (PLE) (supercritical and subcritical fluid extraction—SFE, enhanced solvent extraction (ESE)—involves the use of mixtures water or organic solvents with CO_2_ as solvents, accelerated solvent extraction—ASE), microwave-assisted methods (MAE) and ultrasound-assisted methods (UAM), alongside with non-conventional ohmic technologies (technology relies on ohmic heating by passing electrical current through materials, instead of conductive heat transfer) such as pulsed electric field (PEF) and high voltage electric discharge (HVED), and they can be successfully used for the recovery of antioxidants from agro-industrial by-products. These modern and advantageous techniques require less solvent and energy consumption, and provide enhanced yields of recovery of active compounds [35,36,37,38]. Another innovative technique which can be used in the case of antioxidant recovery is solid-liquid dynamic extraction (RSLDE) by using Naviglio extractor which finds application in various sectors, such as the pharmaceutical, cosmetic, herbal, food and beverage sectors [39]. For this procedure the advantages are working at room temperature, the possibility to recover compounds sensible to the temperature, the principle of the method being based on generating of a gradient pressure between the inner and the outlet of solid matrix [40].

All of these modern techniques have a lot of advantages: increased selectivity in recovering different classes of compounds, small amounts of solvents used, decreased extraction time and less amounts of remaining wastes. However, the major drawbacks appearing during industrial scaling up is the cost of the equipment and its maintenance, the use of adjacent installations needed to provide solvents and other necessary conditions for a proper process at large scale. On the other hand, very good optimization of the parameters is easy to obtain in reproducible conditions, which is often different from laboratory scale.

In case of pressurized liquid extraction (PLE), parameters such as pressure, time, use of co-solvent, etc. are crucial in order to optimize the method and do not affect compounds extracted [41]. PLE is suitable for recovery of non-polar, polar and semi-polar compounds from different matrices of by-products, such as cereals [42], wine making wastes [43], fish industry [44], olives [45] or fruits [46]. However, in this type of extraction, undesirable compounds can be generated (e.g., hydroxymethylfurfural) [47]. The phenomenon can be avoided through the addition of increased amounts of solvents, which increases the temperature of extraction [48]. One of the advantages of PLE is represented by the selective recovery of target compounds. Pereira and coworkers obtained anthocyanin-rich fraction separately from other phenolic compounds with application of PLE [49]. Application of enzymatic pretreatment can also increase the yield of recovery, even few times (e.g., for pomegranate peels) [50]. PLE is also used as a pre-treatment for other techniques [51] or as a complementary technique [52]. The complementary use of PLE enables to improve the yield of recovery and to shorten the extraction time [53].

The use of ultrasounds can facilitate the recovery of antioxidants with high reproducibility and low solvent consumption [30]. Ultrasound-assisted extraction (UAE) was presented as a “green” and safe technology, promoting the release of extractable compounds (like polyphenols and pectins) from fruit peel wastes [54], vegetables [55], or wine industry wastes [56].

Microwave-assisted extraction (MAE) utilizes high frequency, non-ionizing electromagnetic waves which can facilitate the extraction due to a highly localized temperature and pressure. These changed conditions results in the reduction of extraction time and solvent consumption [57]; however, the used solvents have to be permanent dipoles. The use of solvent mixtures extends MAE applications [58,59] to different types of agro-food by-products, such as brans [60], peels [61], seeds [62], wine shoots [63], etc.

Sometimes solvent extraction techniques are not economically feasible and non-conventional techniques like pulsed electric field (PEF) and high voltage electric discharge (HVED) emerged, in order to obtain more efficient and sustainable processes [64,65]. Application of non-conventional techniques strongly depends on the nature of the vegetal material. HVED cannot perform a selective extraction of target compounds due to the total damage of the cell wall [66], whereas PEF is a more selective technique and is applicable to soft tissue matrixes, such as peels [67]. These electrotechnologies can be suitable for different types of agro-food side-streams at industrial levels, such as wine making industry [68], fruits industry [69], or oil industry [35].

In case of isolation and purification of compounds, classical methods such as adsorption on resins, activated carbons or polysaccharide-based materials, membrane processes, nanofiltration or equipment-based methods (counter current chromatography—CCC and centrifugal partition chromatography—CPC) can be used. The first listed have simple design, easy operation and scale-up, and the used materials can be regenerated and re-utilized [70]. The use of these methods is “*a must*” in the recovery of antioxidants from agro-industrial side-streams, such as olive mill waste [71], cereals extracts [72], or fish industry [73].

### 2.2. Recovery of Antioxidant Compounds from Edible Oil Industry Wastes

The edible oil manufacturing processes generate a substantial amount of side streams, which can be further utilized in order to recover different target compounds or as an energy source (some examples presented in Table 2). It is estimated that in manufacturing and processing the vegetable and animal oils, 3.9% represents the remains materials, to be named wastes [74].

The palm fruit is in 10 wt% transformed into oil, the rest being a waste [75]. The kernels, fiber, shells, leaves etc. are waste rich in tocopherols and tocotrienols, humic acid and fulvic acid-like components [76]. The palm fruit is world’s highest yielding oil crop, with eight times higher productivity than rapeseed and six times higher than soybean [77]. The oil is extracted from palm mesocarp and have different composition than oil extracted from kernels. Palm oil industry generates an approximately 94.1 million tones (wet weight) of solid residues [78]. Aside from the waste generated by the palm oil mills, there are the wastes from the plantations which are lignocellulosic-based and can be used as a source of energy [75].

Cardenas-Toro and coworkers established optimal parameters for the recovery of carotenoids from pressed palm fibers applying pressurized liquid extraction (PLE). Authors investigated the influence of solvent, pressure, temperature and flow rate. Using a PLE *homemade* equipment (consisting of a HPLC pump, electrical heating jacket, stop valves and a back-pressure valve) and a Soxhlet apparatus, they obtained better results than for the percolation method [79]. The carotenoid yield obtained using PLE was higher than the obtained by Soxhlet method and percolation (305 ± 18 μg α-carotene/g extract, 142 ± 13 μg α-carotene/g, respectively 79 ± 9 μg α-carotene/g extract). In another study published in 2016, the influence of temperature and pressure were studied for different solvents using the pressurized liquid extraction method (CO_2_ and compressed liquefied petroleum gas) [80]. Extraction yield using supercritical CO_2_ was three times higher than the yield using compressed liquefied petroleum gas, and the properties of the obtained compounds (α-tocopherol, squalene, β-carotene and β-sitosterol) were enhanced in the same condition (CO_2_ as a solvent).

For ultrasound-assisted extraction, the influence of ultrasound intensity and pulse cycle was investigated by Dal Prá and coworkers for the same type of waste, using a central composite rotational design [81]. They recovered β-sitosterol, α-tocopherol, squalene, phenolic compounds and carotene with antioxidant activity and sun protection factor, proposing polar solvents as promising candidates for recovery of target compounds with further use in cosmetic and pharmaceutical formulations.

Olive oil industry is an increasing one with 3.2 million ton in 2015/2016, where EU contributed the most at 2.3 million ton [82]. The conventional olive oil production yields are 20% of olive oil, and 30% of solid residues (such as pomace, kernel, leaves, etc.) [83]. A rich group of antioxidant phenolic compounds classified in four subgroups (phenolic acids and alcohols, lignans and flavones) can be recovered from olive wastes [84].

The recovery of antioxidants from olive leaves was performed by Lama-Muñoz through PLE. The extraction procedure was performed in static mode using a hydroalcoholic mixture as a solvent, and all parameters (temperature, pressure, time, concentration of solvent) were optimized for enhancement of recovery yield for target compounds. The authors obtained oleuropein and luteolin-7-*O*-glucoside through accelerated solvent extraction (extraction method from PLE category) with an increased yield of recovery comparing to classical maceration (27.01 g/kg of dry leaf for PLE method and 63.35 g/kg of dry leaf for classical maceration for oleuropein, respectively 1.18 and 2.74 g/kg for luteolin-7-*O*-glucoside) [85].

Martínez-Patiño and coworkers recovered phenolic compounds and flavonoids from olive tree pruning biomass (old branches) and olive milled leaves using UAE. They established different values of studied parameters: ethanol/water ratio (1/4, 1/1, 4/1), amplitude percentage (30, 50, 70%) and ultrasonication time (5, 10, 15 min) [86]. The obtained results showed that ethanol concentration had the greatest impact on all variables studied for both olive tree pruning biomass (OTP) and olive mill leaves (OML), followed by amplitude and ultrasonication time (ethanol concentration between 54.0 and 55.8% in the case of olive tree pruning biomass and between 50.0 and 53.9% for olive milled leaves).

Xie et al. recovered hydroxytyrosol, maslinic acid and oleanolic acid from olive pomace using UAE and MAE comparative to classical solvent extraction. In order to optimize the extraction conditions in MAE, extraction time was established at 3, 5, 10, 20, 30 and 40 min, microwave power at 100, 200, 400, 600, 800 and 1000 W and extraction temperature 30, 40, 50, 60, 70 and 80 °C. For ultrasound extraction the following parameters were evaluated: 30%, 40%, 50%, 60%, 70%, 80%, 90%, and 100% of the total ultrasound power output (1200 W). Ethanol and hydroalcoholic mixtures were used indifferent temperatures and times for classical solvent extraction. Due to the highest rate of mass and heat transfer, UAE presented the best performance of the extraction efficiency compared with SE and MAE in this experimental case (55.1 mg/g hydroxytyrosol, 381.2 mg/g maslinic acid, 29.8 mg/g oleanolic acid). Other parameters optimized for those extractions was liquid/solid ratio. Authors concluded that in this case, the recovery yield for phenolic compounds was increased in ultrasound extraction comparing to microwave and SE techniques (for hydroxytyrosol 49.3 mg/g, respectively 53.2 mg/g, for maslinic acid 288.2 mg/g, respectively 356.0 mg/g and oleanolic acid 21.5 mg/g, respectively 26.3 mg/g). This was achieved as a consequence of the damage of the plant cell walls and increased amounts of released target compounds [87]. Issaoui and coworkers recovered triterpene (α-tocopherol and squalene) from olive leaves and bark using supercritical fluid extraction. Parameters were optimized especially due to the high solubility of different compounds in the CO_2_, not only for obtaining increased yields of recovery. The best outcome was observed for a pressure of 90 bars [88].

Combination of enzymatic catalysis assisted by ultrasonication offers the possibility to recover target compounds due to the selective nature of enzymes being ecofriendly processes. Ultrasound-assisted enzyme hydrolysis (UAEH) can be performed in order to obtain phenolic compounds from olive pomace. Wang et al. obtained an increased yield of antioxidant recovery compounds (4%), through the optimized method at pH 5.75, time 40 min and a temperature of 55 °C. This method was more efficient than ultrasound treatment alone [89].

Another important edible source in this industrial sector is sunflower, which is the third most important oilseed crop [90]. Food and Agriculture Organization of the United Nations (FAO) forecasted world production of sunflower oil to amount to around 22.4 million tons toward 2050, so there is an intrinsic prediction regarding the amounts of wastes. The wastes resulting from oil extraction are a valuable by-product with high protein contents (40% to 50%) and usually are used as ruminant feed. Also, these wastes are rich in phenolic compounds from which the most frequently is chlorogenic acid [91]. Diterpenoids, flavonoids and lignans with antioxidant capacity can be obtained from sunflower leaves using pressurized liquid extraction (PLE) and enhanced solvent extraction (ESE) in a cascade approach [92]. Enhanced solvent extraction (ESE) is from the PLE category and involves the use of mixtures water or organic solvents with CO_2_ as solvents. In the first stage, PLE was used with critical CO_2_ as a solvent, and the polarity of solvent was changed in the second stage of extraction (ESE with a CO_2_/EtOH/H_2_O mixture as a solvent) for the enhancement of the extraction yields of polar compounds. Using only PLE recovery yield is 0.91%, but when the techniques are used in cascade, the recovery yield is increased (up to 38%) [92].

In developed countries the demand for different types of oils is increased, and the requirements are vast. Rapeseed (*Brassica napus* L.)—rich in protein and polyphenols [93], is one of the major plant sources of oil, usually used as food and raw material for biodiesel production. When it is grown with final application biodiesel, rapeseed can be grown on polluted soils, and the wastes are not suitable for other applications, such as recovery target compounds for human or animal consumption [94]. Until the appearance of the possibilities to recover target compounds, these wastes were used as feed or compost. Electro-technologies were successfully used in the recovery of proteins, polyphenols and isothiocyanates from rapeseed press-cake [95]. Authors evaluated effects of the *liquid-to-solid* ratio and HVED treatments of raw materials influencing the efficiency. Starting from a temperature of 20 °C, the experiment never exceeded 35 °C, in order to do not affect the properties of the target compounds, HVED being a technology suitable for the recovery of antioxidants, if it is optimized specifically for the raw material. For recovery of polyphenols, the optimal energy is decreased for rapeseed press-cake, then in the case of rapeseeds.

Pumpkin is an edible plant which can produce large amounts of waste in agro-food sector, rich in compounds with therapeutic properties (antioxidant, antidiabetic, cardioprotective, etc.), especially used in Europe, USA, Canada, and China [96]. For the recovery of antioxidants from these wastes, advanced techniques can be successfully applied. Ultrasonic-assisted extraction (UAE) and microwave-assisted extraction (MAE) were used by Ferreira and coworkers for the recovery of phenolic compounds from pumpkin seeds, in a comparative study with the classical reflux method [97]. MAE under subcritical conditions was also used in this experiment. In terms of the yield of recovery, the differences for all methods used were observed: UAE gave the most promising results (for lipophilic and hydrophilic compounds), whereas MAE under subcritical conditions provided better results for the recovery of phenolic antioxidant compounds using ethanol and water as solvents (with a double amount of phenolic compounds recovered).

Flax (*Linum usitatissimum* L.) is a good source of edible oil rich in omega-3 fatty acids, with important by-products such as pomace, fibers and shives. The significant growers are Canada (412,000 ha), followed by Russian Federation (410,000 ha) and Kazakhstan (384,300 ha) [98]. For the recovery of flaxseed active compounds, such as tocopherols, polyphenols and phytosterols, pulsed electric field technologies can be applied. Investigations related to operational parameters (the electric field intensity, input treatment energy, solvent, pH) revealed that best yield was obtained with up to 80% of polyphenols with the rehydration of the raw material before extraction [62].

### 2.3. Recovery of Antioxidant Compounds from Fruits and Vegetable Wastes

In this sector, researchers have different opinions regarding the terms and definitions. This concept is being very controversial: for some authors, the term “fruit and vegetable waste” represent the inedible parts of fruits or vegetables that are obtained during collection, handling, transportation and processing [99]; for other authors, this represents “*the decrease in edible food mass throughout the part of the supply chain that specifically leads from raw material to food for human consumption*” [100]. However we define those wastes, this industry presents a large amount of by-products. Fruit and vegetable production have been increased, reaching approximatively 0.9 billion tons of fruit and more than 1 billion tons of vegetables, in 2017 [101]. In the fruits sector, the most produced are citrus, watermelons, banana, apples, and grapes, while for vegetables are potatoes, tomatoes, onions, cucumbers and cabbages [102]. According to FAO statistics, a large amount of foods ends up in the garbage (approx. 1.3 billion tons) distributed on different sectors: from fruits and vegetable industry resulting 66% by weight of total food losses, roots and tubers account for 44 and 20% by weight [103,104].

Also, there is the category of “sub-standard products”, which represent the fruits and vegetables that have small dimensions and do not fulfill quality standards, regulated by EC Regulation No 1221/2008, and are used for some derivatives, like juices, vinegar, etc., generating in turn different by-products.

The inedible wastes resulting from processing of fruits and vegetables is represented by peels, pomaces, seed and depending on source, have different ratios: for bananas, pineapples and citrus (25–46%), apples (12%), cauliflower (43%), carrots (20%), and garlic (22%) [105]. Due to the large amounts of moisture, these residues are perishable [106] and implies various difficulties in storage [107], being necessary a pre-treatment for further utilization for recovery of target compounds [108]. Side streams from fruits and vegetable sector represent a rich source in bioactive compounds, especially antioxidants (some examples presented in Table 3). For these wastes, antioxidants, especially phenolic compounds composition, can be modified by fermentative processes which can take place in large amounts of waste. Some authors suggested that fermentation increases the phenolic content [109,110], while others observed a decrease for antioxidant concentration [111], or even a compound degradation [112]. So, a proper and rapid extraction treatment is necessary to recover antioxidants from wastes (pomace, peels, etc.).

#### 2.3.1. Fruits Wastes

For recovery of target compounds with increased yields, different extraction methods can be applied themselves or in combination. Nile and coworkers have exploited apple pomace in order to obtain phenolic compounds and triterpenic acids through a classical centrifugation method, removing the solvent with vacuum [113]. The influence of the solvent on the chemical composition and yield of recovery for phenolic and triterpenic compounds was investigated simultaneously. The methanol and ethanol fractions presented highest antioxidant potential with potential tyrosinase, xanthine oxidase and urease inhibition. Chandrasekar optimized MAE of antioxidants from apple pomace and developed conditions were independent of apple variety. The authors proposed MAE as an interesting extraction technique which can be industrially scaled up for this type of wastes. [114].

Supercritical CO_2_ extraction was applied for mango peels by del Pilar Sánchez-Camargo and coworkers. Antioxidants (carotene) were obtained using two drying methods (freeze-drying and air-drying) as a pretreatment step. The optimal conditions were also established using a Box-Behnken design. Using freeze-drying pretreatment, the amount of antioxidants obtained was increased (2.01 ± 0.05 mg all-trans-β-carotene equivalent g^−1^ dried mango peel compared with 1.22 ± 0.02). Although the freeze-drying treatment was more effective to preserve active compounds against oxidation than air drying, this last technique becomes more attractive due to its simplicity and speed [115].

In order to enhance limonene recovery, the influence of several solvents was investigated in the case of orange peel waste valorization [116]. Solvents were selected considering Environmental, Health and Safety parameters so as to replace hexane [117] and a COnductor-like Screening MOdel for Real Solvents (COSMO-RS) was used [118] to rank the solvent performance. Also, extraction time, temperature and solid to liquid ratio were assessed: for T = 70 °C, t = 150 min, S/L = 1:10 the recovery yield was increased up to 80% for cyclopentyl methyl ether and 2-methyl-tetrahydrofuran, instead of hexane.

Cocoa shells are valuable sources for recovery catechins, epicatechins, theobromine and caffeine through ultrasound-assisted extraction (UAE) and hydrodynamic cavitation (HC) [119]. The recovery yield was enhanced in the case of HC with a ternary mixture of solvents (hexane, ethanol and water) (14.6% compared with 13.9%). Also, defatting pre-treatment increased recovery yields through, the extraction time decreasing to half. When some extracting parameters are increased, for some compound recovery yield decreases. This is the case of procyanidin B2 from cocoa shells for which recover yields decreases in the case of increasing temperature and extraction time for PLE [120].

#### 2.3.2. Vegetable Wastes

For the recovery antioxidants from vegetable wastes, the same methods as in the case of fruits can be applied. Yilmaz et al., revealed that using ultrasound extraction method increased amounts of lycopene from tomato seeds can be obtained (from 52.21 mg/kg to 70.10 mg/kg), at an extraction time of 10 min, instead of 20 min used for a classical method (maceration) [121]. Microwave-assisted extraction applied for tomato seeds increases the recovery yield of lycopene and assure the retention of all-trans-lycopene by retarding the isomerization to its cis-isomers [122].

In a more complex study, Coelho and collaborators optimized ohmic technologies for the extraction of antioxidants from tomato seeds, in order to identify the presence of non-thermal effects on the extraction process [123]. The optimal conditions were established at 70 °C for 15 min using 70% ethanol, rutin being recovered in a percentage of 77% higher than control samples. The extraction process permitted a permeabilization of the cells that can help obtain a selective extraction process of target components from tomato by-products.

Combining an eco-friendly method, as subcritical water extraction with physical pretreatment by intense pulsed light the recovery of quercetin from onion waste was performed [124]. The pretreatment increased the recovered amount of quercetin in optimal conditions. The method of pretreatment and extraction was also used at larger pilot scale in order to confirm the possibility of practical industrial application. The intense pulsed light can damage the cell walls releasing target compounds being a promising pretreatment technology. Comparative to other methods used as pretreatment, intense pulsed light could reduce the loss of nutrients and the energy cost, due to very short treatment times [125]. For pressurized liquid extraction with CO_2_ as non-polar solvent, a modifier such as ethanol is needed to recover lutein and chlorophyll from spinach by-products [126]. Compared to aqueous ethanol extraction, through this technology increased yields of recovery for target compounds are obtained.

Another interesting aspect of recovery antioxidant from agro-industrial side streams is the use of vegetable oil as a solvent, in order to obtain oils enriched with antioxidants. Due to great solubility of carotenoids in edible oil, sunflower oil was used as a solvent for ultrasound-assisted extraction of carotenoids from pomegranate wastes [127]. The oil plays a role of a barrier against oxygen, and the degradation rate of the carotenoids in the extract is decreased. Highest carotenoids yield (26.3 μg/g waste) was obtained by extraction with sunflower oil in comparison with soy oil, coconut oil, and rice bran oil, approximately 93.8% of the total carotenoids present in the waste material being extracted.

### 2.4. Recovery of Antioxidants Compounds from other Different Industries

Other industries from agro-food sector, which can produce large amounts of side streams are animal origin derived products as meat (with side streams bones, tendons, skin, parts of the gastrointestinal tract and other internal organs, and blood), and fish. They have a large annual production of 263 million tones and 128 million of tones, respectively [128]. In the fish industry, more than 50% is considered to be waste [129]. Beside the losses resulted from the production processes, round 88 million tons of food (meat, fish and other animal origin products) are wasted annually only in the EU, with associated costs estimated at 143 billion € [130]. Even though the losses in animal origin products industry are in general less than in agro-industry (20% comparative with crops production –30%), meat waste has the highest negative environmental impacts estimated by greenhouse emissions [131]. In this respect, the recovery of active compounds from wastes and their further utilization is a solution mitigating negative environmental impact (Table 4).

By-products from the fish and meat industry are rich sources of important biologically active molecules, such as amino acids, peptides [132], proteins [133], proteolytic enzymes, collagen, gelatin, keratin, chitosan, fatty acids and peptone [1]. Some target compounds have antioxidant properties (amino acids like histidine and aromatic amino acids like phenylalanine) due to quenching metal ions and reactive oxygen species.

For the extraction methods of target compounds from these types of materials more attention must be payed to other details, than in the case of plant-based materials, due to their complex matrix and the possibility to obtain more diversified categories of wastes (skin, blood, shells, etc.). Most of the studies describes a combination of thermal, mechanical, chemical and enzymatic methods, such as blending followed by extraction with alkali/acid and hydrolysis with pepsin/pancreatin for the obtaining of peptides from spent hen meat hydrolysate [134] or homogenization followed by enzymatic hydrolysis [135], homogenization, hydrolysis, and ultrafiltration for obtaining carnosine from chicken meat [136].

However, modern approaches are needed for the recovery of antioxidant target compounds in a sustainable development [137]. In subcritical water extraction method, the interactions between water and non-polar substances occur due to the changes in electrochemical properties. Thus, binding force is decreased and organic matter is more easily dissolved and hydrolyzed resulting in better release and recovery of target compounds. This is the case of proteins which can be transformed into peptides and free amino acids that can be easily recovered by conventional techniques such as ultrafiltration or spray-drying [138]. For protein extraction, chemical and enzymatic hydrolysis can be performed using on one hand hazardous chemicals, on the other hand expensive reagents such as enzymes, and all the process parameters must be controlled very rigorously [138]. Asaduzzaman and Chun studied the recovery of amino acids and peptides from squid muscle, by subcritical water extraction. The optimal recovery conditions were established 250 °C for amino acids, 160 °C for peptides, and some complex amino acids were degraded at 250 °C [139]. Zhu et al. recovered amino acids from poultry wastes (chicken intestine), with a proper establishment of the process parameters (reaction temperature 533 K, reaction time 28 min and H_2_SO_4_ concentration in reactant system 0.02%) [140].

In order to achieve an easier recovery, ohmic technologies can be used for denaturation of proteins and changing the morphology of the produced protein aggregates. This is the case of whey proteins which change their behavior under moderate electric fields and controlled temperature [141]. For proteins from muscle, sets of high voltage short pulses and by low voltage long pulses are needed in order to their recovery, due to the creation of more pores from which the liquid may come out easily from the biomass [142].

Enzymatic antioxidants can be successfully obtained from brewery industry, where, as by-products are considered spent grains, spent hops and surplus yeast. Usually, these by-products having a high content of proteins, are used to local dairy farmers to be used as cattle feed, or simply as a land fill [143]. From this type of material recovery of enzymatic antioxidants can be made through enzymatic hydrolysis with exogenous enzymes, the process having higher specificity than conventional processes [144]. Brewing materials and beer are also rich in polyphenols derived from barley malt and hops [145] which can be recovered through solid-liquid extraction techniques [146].

The concept of biorefinery and transforming biomass into value added products can also be applied to algae biomass [147] which is a rich source of active compounds [148]. In order to obtain antioxidants extraction techniques can be applied for many edible species, Fucoidan can be obtained from brown algae through MAE [149,150] and supercritical fluid extraction [151]. Phlorotannins were extracted from *Saccharina japonica* Aresch through optimized MAE [152], phytosterols (fucosterol, 24-methylenecholesterol,) and phytol from edible marine algae by MAE coupled with high-speed counter-current chromatography [153]. For tocopherol recovery from *Porphyridium cruentum* (S.F.Gray) Nägeli UAE in a proper mixture of solvents was applied [154]. Carotenoids were obtained through electro-technologies [155]. Each of the extraction methods present advantages and disadvantages, however the researches on efficient processing techniques were performed just at the laboratory scale [156].

## 3. Potential Applications of Antioxidants Recovered from Food Waste and by-Products

Besides colorants, preservatives or texturizing agents produced in circular economy, antioxidants find its second application mainly as food additives [157], functional foods and nutra-/pharmaceuticals [158], but recently also as functional cosmetics (cosmeceuticals as anti-aging products, whitening products and sunscreens), beauty products (makeup products) [159,160] and biopackaging [161,162,163]. Plant derived antioxidants are compatible with food products and were already confirmed to be helpful in shelf life extension. Extracts from prune, grape, bearberry, grape seeds, rosemary, clove and mango shell are effective in conservation of meat and meat products, significantly decreasing lipid oxidation comparing to control under refrigeration conditions with greater effect in raw meats than in cooked ones [164,165,166,167,168,169,170]. Potato peel, sugar beet pulp and rosemary successfully control the oxidation in sunflower oil and soybean oil [171,172]. The bakery products enriched by plant extracts or powdered plant materials are appreciated for their taste. Nevertheless, the addition of lavender or *Melissa* waste, grape or moringa extracts results in the extension of enjoyable consumption of breads and cookies [173,174,175].

The reuse of food waste and food by-products within the same industry facilitates the waste management and lowers financial outlays spent for eco-friendly production. The eco-aspect is furthermore covered by production of bio-packaging. The focus is placed on development of active packaging from natural biodegradable polymers supported by plant antioxidants. Soy protein isolate or fish gelatin may be transformed into films enriched with mango kernel extracts, licorice residue extract, pomegranate peel powder or pine bark extract to name just a few, resulting in functional release of antioxidants over time and delayed spoilage of covered products [161,162,163,176,177]. Besides film wrappings, biodegradable containers being in direct contact with foods are also designed. Incorporation of herbal antioxidants is especially advantageous in fatty food applications. Such containers enriched with e.g., achiote or yerba mate decreases oxidation processes within food, hence prevent the changes in sensorial and nutritional product characteristics [178,179,180].

Circulation of redundant food biomass within food industry is ensured likewise through utilization of secondary food products into functional foods. High fiber waste as pineapple peel and core, mango rind, cactus pear peel or broken rice is easily introduced into cereal bars or cakes [181,182]. Red fruits concentrates are basis of sports drinks rich in easily available energy from glucose, fructose, sucrose or maltodextrin/glucose polymers. Polyphenols from various berries, cocoa, ginger, vegetables and seeds are often found in fortified antioxidant functional beverages, which are also available as fermented products [183]. Since healthy eating and healthy lifestyle attracts a lot of attention in a modern society, functional foods are a well-established avenue for introduction of recovered bioactive compounds.

Healthy products are usually associated with natural or ecological as well. This brings a current eco-beauty movement, based on re-utilization of food wastes into daily cosmetics showing new applications of food derived antioxidants. Cosmetic companies participate is so-called “circular beauty” by introducing to the markets fast-growing number of products including repurposed food waste. The food and drink manufacturers serve directly as a source ingredient which are transformed into: coffee scrubs made from reclaimed coffee grounds; range of hair products and hair dyes rich in anthocyanins obtained from blackcurrant residues; lip balms and colors using processed fruit waste; hand soap and candles from cooking grease [159,160]. The added value products obtained from agro-industrial side streaming supply also the production of cosmeceuticals. These skin “quasi drugs” are aimed to exert anti-aging, whitening or sunscreen effects which are based mainly on the action of plant antioxidants. Market products rich in resveratrol, arbutin, organic acids, rutin, but also vegetable oils, create the demand for pure compounds obtained in the eco-friendly and sustainable way. Similar market demand is still factual in case of nutraceuticals and herbal drugs, which are frequently composed of blended plant extracts or purified compounds [168].

The proved beneficial effects exerted by natural antioxidants are observed mainly in prevention of cardiovascular disease and cancer and mitigation of diabetes complications. The inhibition of low-density lipoprotein oxidation underlies the successful delay of atherosclerosis development. Action of antioxidants within plasma and mitochondrial membranes results in beneficial effects on platelet aggregation. Additional scavenging of peroxynitrite generated by the reaction of nitric oxide and superoxide anion ensures the proper amount of nitric oxide for maintenance of flexible blood vessels for normal blood flow [184,185]. The same action of antioxidants in endothelium of veins is beneficial in management of consequences of diabetes. Since higher levels of reactive oxygen species generated in diabetes were proved by clinical evidence, the deleterious effects of this disease are usually associated to oxidation. High blood glucose levels additionally promote auto-oxidation of glucose to form free radicals. Supplementation with antioxidants helps to protect beta cells of the pancreas and make them function correctly [186]. What is more, lipophilic antioxidants, were shown to selectively regulate peroxisome proliferator-activated receptors, being a ligand-regulated transcription factor playing essential role in energy metabolism [187]. This action of antioxidants on molecular level results in improvement of body glucose utilization and insulin sensitivity [185,188,189]. Antioxidants were also shown to have ability to modulate molecular mechanisms in cancer cells. Their cancer-preventive and -therapeutic effects result from suppression of inflammation, oxidative stress, proliferation and angiogenesis [190]. Positive effects in cancer treatment were frequently observed for fruit antioxidants in animal models and human clinical trials [191].

Some examples of antioxidants from the above-described industries and their applications are listed in Table 5.

## 4. Conclusions and Perspectives

Advanced extraction techniques are promising tools to recovery of antioxidants from agro-industrial side streams. The nature of waste materials is very heterogenous, so there is not a general strategy applicable, in order to recovery active compounds. The attention of scientific community is focused on optimizing extraction techniques, in order to obtain increased yields of production in an ecofriendly and economic way. Recovering techniques must be adapted to raw material and, in the same time to final application. The cost of the process related to the value of recovered compounds must be evaluated, especially in industrial scale up cases. In order to answer the short-comings of classical extraction techniques, advanced techniques emerged, achieving more efficient and sustainable processes.

From all the described advanced techniques, electro technologies seem to be the most appropriate to term “green”. They are applicable, for different types of agro-food side streams at industrial levels, from wine making industry to oil industry. However, due to the equipment requirements and maintenance costs, they are not ready to be scaled up at industrial level even though they present enhanced efficiency in the recovery of antioxidants comparing to other techniques. For the extraction methods some conditions must be fulfilled to be economically feasible: target molecules must have market potential and the ratio production costs-benefit must be profitable.

Future research directions for a proper management of these waste are conducted also, in term of pretreatment, in order to extend their durability, minimize the loss of bioactive compounds and reduce environmental pollution. The development of tandem processes (more than two techniques applied for recovery target compounds) can offer significant advantages in terms of selectivity, decreasing energy consumption and increased yield of recovery bioactive compounds, providing an essential contribution to the process intensification strategy.

## Figures and Tables

**Table 1:** Examples of different antioxidants from agro-industrial side-streams.

Compound Group	Source	Extracted Compounds	Ref.
Phenolic compounds	Apple seeds	Phloridzin, ellagic acid, caffeic acid, ferrulic acid, protocatechuic acid, gallic acid	[12]
Phenolic compounds	Avocado seeds	Procyanidin B2, epicatechin, rans-5-*O*-caffeoyl-d-quinic acid, procyanidin B1, catechin	[13]
Phenolic compounds	Rapeseed cake	Sinapine, sinapic acid and canolol	[14]
Phenolic compounds	Citrus peel	Total phenolic content	[15]
Phenolic compounds	Coconut shell	Total phenolic content	[16]
Phenolic compounds	Grape marc, Orange peel, Strawberry, Citrus pulp Camelina cake	Total phenolic content	[17]
Phenolic compounds	Black currant Sea buckthorn	Delphinidin 3-*O*-rutinoside, delphinidin 3-*O*-glucoside, cyanidin 3-*O*-rutinoside, cyaniding-3-*O*-glucoside, ellagitannins, proanthocyanidins, *p*-coumaric acid, caffeic acid-hexosides, coumaroylquinic acid-hexosides, vanillic acid-hexoside, (+)-Catechin, (−)-epicatechin, Quercetin 3-*O*-rutinoside, 3-*O*-glucoside, and 3-*O*-(6′′-malonyl)-glucoside	[18]
Phenolic acids, flavonoids	Grape skin	Gallic acid, caffeic acid, epicatechin, *p*-coumaric acid, rutin, catechin gallate	[19]
Flavonols	Pistachio hulls	Gallic acid, penta-*O*-galloyl-β-d-glucose, anacardic acid	[20]
Flavonoids, carotenoids	Passion fruit peel	β-carotene, provitamin A, quercetin, lycopene	[21]
Carotene	Carrot pomace	α- and β-carotene	[22]
Lycopene	Tomato peel	Lycopene	[23]
Non phenolic compounds	Lettuce	Ascorbic acid	[24]
Non phenolic compounds	Sugarcane molasses	Pullulan	[25]
Non phenolic compounds	Rice bran oil	Tocopherol	[26]

**Table 2:** Some examples of recovery of antioxidant compounds from edible oil industry wastes ^1^.

Waste	Extraction Method	Optimized Extraction Conditions	Obtained Compounds	Antioxidant Assay	Ref.
Flaxseed hulls	PEF	Electrode area (cm^2^)—95 pulse length (µs)—10 Temperature (°C)—20 Electric field (kV/cm)—20	Tocopherols, polyphenols, phytosterols	-	[62]
Palm pressed fibers	PLE, Sx, Pc	Temperature (°C)—35, 35, 78.4 Flow rate (g/min) 2.4 Pressure (Mpa)—0.1; 0.1; 4	Carotenoids	-	[79]
Palm pressed fiber	PLE	Solvents: CO_2_ and compressed liquefied petroleum gas Temperature (°C)—60 Pressure (MPa)—25.0	β-sitosterol, α-tocopherol, squalene	HPX/XOD	[80]
Palm pressed fiber	UAE	Ultrasound intensity (W.cm^−2^)—120 Pulse cycle 0.4 Temperature (°C)—20	β-sitosterol, α-tocopherol, squalene,	DPPH ABTS	[81]
Olive leaves	ASE	Temperature (°C)—190 Leaf moisture content (%)—5 Aqueous ethanol concentration (%)—80	Oleuropein, Luteolin-7-*O*-glucoside	DPPH	[85]
Olive tree pruning biomass Olive mill leaves	UAE	Power (W)—400 Frequency (kHz)—24. Liquid/solid ratio of extraction (*v*/*w*)—20 mL/g.	Phenolic compounds Flavonoids	DPPH, ABTS, FRAP	[86]
Olive pomace	UAE, MAE, Se	Ethanol concentration (%)—90, Temperature (°C)—50, Time (min)—5 Liquid /solid ratio (mL/g)—30 Ultrasound intensity (W/cm^2^)—135.6 Ultrasound frequency (kHz)—60	Hydroxytyrosol, maslinic acid, oleanolic acid	-	[87]
Olive leaves and tree bark	SCe	Temperature (°C)—60, Pressure (bar)—300	α-tocopherol, squalene	-	[88]
Olive waste	UAEH	Cellulase, pectinase Frequency (kHz)—40 Power (W)—200	Phenolic compounds	DPPH, ABTS, FRAP	[89]
Sunflower leaves	PLE, ESE	CO_2_ and mixture of solvents (ethanol in water from 0 to 100%) Pressure (bar)—400 Temperature (°C)—55	Diterpenoids, flavonoids	-	[92]
Rapeseed press-cake	HVED	High voltage pulsed power (kV)—40 Intensity (kA)—10 Needle diameter (mm)—10	Protein, polyphenols and isothiocyanates	TEAC	[95]
Pumpkin seeds	UAE MAE	Frequency (GHz)—2.45 Ethanol concentration (%)—60 Time (min)—20 UAE-EtOH—60% UAE-hex/EtOH/ H_2_O—30:49:21%	Phenolic compounds	DPPH	[97]

^1^ Where: ABTS—2,2′-azino-bis(3-ethylbenzothiazoline-6-sulphonic acid; ASE—accelerated solvent extraction; DPPH (assay)—2,2-diphenyl-1-picrylhydrazyl; ESE—enhanced solvent extraction; EtOH—ethanol; FRAP (assay)—ferric reducing ability of plasma; hex—hexane; HVED—high voltage electric discharge; MAE—microwave-assisted extraction; Pc—percolation; PEF—pulsed electric fields; PLE—pressurized liquid extraction; SCe—supercritical extraction; Se—solvent extraction; Sx—Soxhlet extraction; TEAC (assay)—Trolox equivalent antioxidant capacity; UAE—ultrasound-assisted extraction; UAEH—ultrasound-assisted enzyme hydrolysis; HPX/XOD—Hypoxanthine/xanthine oxidase system (superoxide radical scavenging activity).

**Table 3:** Some examples of recovery of antioxidant compounds from fruits and vegetable wastes ^1^.

Waste	Extraction Method	Optimized Extraction Conditions	Obtained Compounds	Antioxidant Assay	Ref.
Apple pomace	Cec	Methanol, ethanol and ethyl acetate	Phenolic compounds and triterpenic acids	DPPH, FRAP, ABTS	[113]
Apple pomace	MAE	Solvent—70% acetone and 60% ethanol, Microwave power (W)—735, Solvent volume to sample ratio (mL/g)—5.65 Time (s)—149	Phenolic compounds	DPPH	[114]
Mango peels	ScE	Pressure (MPa)—25.0 Temperature (°C)—60 Solvent—15% *w*/*w* ethanol	Carotene	-	[115]
Orange peel	LSE	Solvent: cyclopentyl methyl ether, ethyl lactate, isopropyl alcohol, polyethylene glycol 300, isopropyl acetate, dimethyl carbonate, methyl ethyl ketone, 2-methyl-tetrahydrofuran and ethyl acetate Temperature (°C)—70 Time (min)—150 Solid -liquid ratio—1:10	Limonene	-	[116]
Cocoa shells	UAE, HC	Hexane, hydro-alcoholic solution (70:30 EtOH/H_2_O) ternary mixture (30:49:21 Hex/EtOH/H_2_O) cycle number 47.1, cycle time (s)—5 residence time (s)—5 total residence time (min)—3.93	Catechins epicatechins theobromine caffeine	DPPH	[119]
Tomato seeds	UAE	Power (W)—90 hexane-acetone-ethanol 2-1-1	Lycopene	-	[121]
Tomato seeds	MAE, OT	Temperature (°C)—70 Time (min)—15 Solvent—70% ethanol	Rutin	-	[123]
Onion waste	SbWE(PT)	Temperature (°C)—145 Time (min)—15 intense pulsed light (V)—1200 Time (s)—60	Quercitin	-	[124]
Pomegra-nate waste	UAE	Temperature (°C)—51.5; Amplitude level—58.8% Solvent—sunflower oil	Carotenoids	-	[127]

^1^ Where: ABTS—2,2′-azino-bis(3-ethylbenzothiazoline-6-sulphonic acid; Cec—classical extraction with centrifugation; DPPH (assay)—2,2-diphenyl-1-picrylhydrazyl; FRAP (assay)—ferric reducing ability of plasma; HC—hydrodynamic cavitation; LSE—liquid solid extraction; MAE—microwave -ssisted extraction; OT—ohmic technologies; SbWE(PT)—Subcritical water extraction with physical pretreatment; ScE—Supercritical extraction; UAE—ultrasound-assisted extraction.

**Table 4:** Some examples of recovery of antioxidant compounds from other different industries wastes ^1^.

Waste	Extraction Method	Optimized Extraction Conditions	Obtained Compounds	Antioxidant Assay	Ref.
Squid muscle	SbWE	Temperature (°C)—250 for aminoacids; 160 for peptides	Amino acids Peptides	ABTS	[139]
Poultry wastes	SbWE	Temperature (K) 533 Reaction time (min)—28 H_2_SO_4_ concentration in reactant system 0.02%.	Amino acids	-	[140]
Waste chicken breast muscle	OT	Sets of high voltage short pulses and by low voltage long pulses Energy (J/g)—38.4 ± 1.2	Proteins	DPPH ABTS	[142]
*Fucus vesiculosus*	MAE	Pressure (psi)—120 Time (min)—1 1g alga/25mL water	Fucoidan	-	[149]
*Saccharina japonica* Aresch	MAE	Solvent: 55% ethanol Irradiation power (W)—400 solid/solvent ratio 1:8; Time (min)—25	Phlorotannins	-	[152]
*Undaria pinnatifida* and *Sargassum fusiforme*	MAE coupled with HSCCC	Solvent: ethanolic KOH solution (1.5 mol/L) Irradiation power (W)—500 Liquid/solid ratio 20:1 Time (min)—20 Revolution speed (RPM)—800	Fucosterol, 24-methylenecholesterol, phytol	-	[153]
*Porphyridium cruentum*	UAE	Solvent: 2mL of ethanol, 10mg ascorbic acid, 3mL of n-hexane, Time (min)—20	Tocopherol	-	[154]
*Nannochlorops* sp.	PEF	The electric field (kV/cm)—20 Consecutive pulses 1–400	Carotenoids	-	[155]

^1^ Where: ABTS—2,2′-azino-bis(3-ethylbenzothiazoline-6-sulphonic acid; DPPH (assay)—2,2-diphenyl-1-picrylhydrazyl; HSCCC—high-speed counter current chromatography; PEF—pulsed electric fields; OT—ohmic technologies; MAE—microwave-assisted extraction; SbWE—subcritical water extraction; UAE—ultrasound-assisted extraction.

**Table 5:** Recovery of antioxidant compounds from agro food side streams and potential applications.

Waste	Active Compounds	Application	Ref.
*Applications of antioxidant compounds recovered from edible oil industry wastes*			
Palm pressed fiber	β-Sitosterol, α-tocopherol, squalene	Cosmetic formulation with high sun protection factor	[81]
Sunflower leaves	Diterpenoids, flavonoids	Natural herbicide	[92]
Sunflower seed	Phenolic compounds	Antioxidant additive for sunflower oil	[192]
Soy bean waste	Proteins	Biopackaging	[193]
Olive waste extract	Phenolic compounds	Food industry (increasing shelf life of meat)	[194]
Olive mill wastes	Phenolic compounds	Food antioxidants	[195]
*Applications of antioxidant compounds from fruits wastes*			
Apple seeds	Phenolic compounds	Food industry	[12]
Berries	Phenolic compounds	Pharmaceutical formulations	[191]
Mango peels	Carotene	Antioxidant additive for edible oil	[115]
Banana peels	Caffeic acid	Cosmetic formulations	[196]
Citrus peels	Phenolic compounds, essential oils and flavonoids	Pharmaceutical formulations	[197]
Citrus wastes	Phenolics and flavonoids	Cosmetic formulations	[198]
Citrus peels	Terpinene, cymene	Pharmaceutical formulations	[199]
Cocoa	Total extract	Larvicidal nanoparticles	[200]
Grape pomace	Phenolic compounds	Food industry	[201]
*Applications of antioxidant from vegetable wastes*			
Tomato wastes	Lycopene	Health related applications	[202]
Beetroot pomace	Betalains	Medicinal and food applications	[203]
Carrot pomace	Carotenoids	Pharmaceutical formulations	[204]
Garlic waste	Ethanolic extract	Food additive to increase products shelf life	[205]
Onion waste	Phenolic compounds	Food industry	[206]
Cauliflower by-products	Isothiocyanates	Food industry	[207]
*Applications of antioxidant compounds from other industries*			
Meat industry wastes	Gelatin Heparin	Pharmaceutical formulations (antioxidant and antihypertensive)	[1]
Algal biomass	Sulfated polysaccharides	Pharmaceutical formulations	[208]
Algal biomass	α-Carnitine	Nutraceutical products	[209]
Squid waste	Astaxanthin	Pharmaceutical industry	[210]
Shrimps shells	Astaxanthin	Food packaging material	[211]
Shrimps shells	Carotenoprotein	Supplementary nutritive feed	[212]
